# Development of a Starvation Response–Based Model and Its Application in Prognostic Assessment of Liver Hepatocellular Carcinoma

**DOI:** 10.1155/mi/8828435

**Published:** 2025-07-07

**Authors:** Xinjun Hu, Yafeng Liu, Shujun Zhang, Kaijie Liu, Xinyu Gu

**Affiliations:** ^1^Department of Infectious Diseases, The First Affiliated Hospital, College of Clinical Medicine, Henan University of Science and Technology, Luoyang 471000, China; ^2^Department of Oncology, The First Affiliated Hospital, College of Clinical Medicine, Henan University of Science and Technology, Luoyang 471000, China

**Keywords:** glycolysis, liver hepatocellular carcinoma, prognosis, RiskScore model, single-cell RNA sequencing, starvation response, transcriptome

## Abstract

**Background:** Hepatocellular carcinoma (LIHC) is a highly prevalent and poorly prognostic malignancy worldwide, and nutrient deprivation in the tumor microenvironment activates the starvation response in tumor cells. Starvation response-related genes (SRRGs) play critical roles in maintaining energy metabolism and promoting tumor development, but their value in prognostic prediction of LIHC has not been clarified.

**Methods:** We based on public databases to obtain transcriptome and single-cell RNA sequencing (scRNA-seq) data for LIHC and SRRG from previous studies. Key modules relevant to SRRGs were identified by weighted gene co-expression network analysis (WGCNA). Functional enrichment analysis was conducted using clusterProfiler R package. Independent prognostic genes were screened to build a RiskScore model and its performance was further verified. The immune microenvironmental profile of patients in different risk groups was assessed using the single-sample gene set enrichment analysis (ssGSEA), MCP-Counter, ESTIMATE, and TIMER algorithms. Seurat package for single-cell profiling and validation of key gene expression based on Huh7 and transformed human liver epithelial-2 (THLE-2) cell lines. The LIHC cell migration and invasion were measured by conducting wound healing and transwell assays.

**Results:** The key module identified by WGCNA showed the strongest correlation with SRRGs and the glycolysis-related SRRGs were mainly enriched in metabolism-correlated pathways. Two protective genes (FBXL5 and PON1) and three risk genes (TFF2, TBC1D30, and SLC2A1) were discovered as the independent prognostic genes for LIHC. Activation of cytokine–cytokine receptor interaction and IL-17 signaling pathway and higher infiltration of immune cells in high-risk group was observed. The five independent prognostic genes were mainly expressed in cancer stem cells and epithelial cells, in particular, *SLC2A1* and *TFF2* were significantly high-expressed in epithelial cells in the tumor group than in nontumor group. *FBXL5* and *PON1* were downregulated, while *TFF2*, *TBC1D30*, and *SLC2A1* were upregulated in LIHC cells. Silencing SLC2A1 significantly inhibited LIHC cell migration and invasion.

**Conclusion:** In this study, we constructed the first risk model based on SRRGs to accurately predict the prognosis of LIHC, which provides a new idea for individualized treatment and targeted intervention.

## 1. Introduction

LIHC accounts for roughly 80% of primary liver cancer cases [1, 2]. Difficulties in the early detection of LIHC are indirectly correlated with an unfavorable 5-year survival rate as low as 18% [3]. Recent statistics showed that LIHC ranks the sixth highest in cancer incidence and the third major cause of cancer deaths [4, 5]. The onset of LIHC is a complex process, which is principally initiated during chronic inflammation such as exposure to infectious pathogens and hazardous substances [6, 7]. At present, early-stage LIHC patients are normally treated by surgical resection and liver transplantation, but generally there is no effective therapy for late-stage LIHC [8]. Previous study showed that only 5%–10% of LIHC patients are eligible for taking surgery. However, postoperative hepatic failure [9] and a 5-year recurrence rate of as high as 70% will seriously affect the survival quality of patients [10]. Currently, tyrosine kinase inhibitors (TKIs), for example, Regorafenib, Sorafenib, and Lenvatinib, are widely used drugs for targeted treatment of advanced LIHC [11], but some patients respond limitedly to TKI therapies [12]. Immunotherapy based on ICIs has also emerged as an alternative option for the management of advanced LIHC patients [13]. However, we still face a lack of accurate molecular biomarkers to improve the diagnosis, immunotherapy response, and prognostic results of LIHC patients.

TME is critically involved in the initiation of LIHC and its development [14]. Rapid division and proliferation of tumor cells shape an adverse TME that is characterized by glucose deprivation and amino acid shortage and most malignancies have the capacity to resist nutrient-deprived conditions in the TME [15]. Tumor cells could not only detect starvation via various proteins (such as GCN2, AMPK, and mTOR) or chromatin modification to promote nutrient absorption, but also flexibly increase key metabolic enzymes in glycolysis pathways to ensure energy supply [16]. Moreover, autophagy is an adaptive behavior and also a protective mechanism against malnutrition stress to adjust tumor cell migration and invasion [17]. The activation of autophagy facilitates the metastasis and glycolysis of LIHC cells through inducing the expression level of MCT1 and stimulating Wnt/β-catenin signaling pathway [18]. Study reported that disrupting the adaptive response of cancer cells may be a promising therapeutic method, for instance, promoting the expression of fructose-1,6-bisphosphatase 1, a downstream gluconeogenic enzyme that can suppress the glycolysis and tumor growth [19]. Nevertheless, study on the molecular mechanism of LIHC progression in response to starvation in the TME is still limited.

Advances in the development of bioinformatics techniques and high-throughput sequencing analysis has enabled the identification of important targets and novel predictive markers [20, 21]. Currently, some researchers have provided important methodological and theoretical basis for developing risk scoring models with starvation response-related genes (SRRGs), contributing to the analysis of immune microenvironment and the validation of key gene functions [22, 23]. We aimed to construct and validate risk models for predicting the prognosis of LIHC patients based on SRRGs (Supporting Information 1: Figure S1). Relevant datasets were collected from the public databases for the current study. Then, an independent RiskScore model was established based on SRRGs and validated. Immune cell infiltration and functional enrichment pathways in different risk groups were compared. Further, a nomogram combining clinicopathological features and the RiskScore was established. The expressions of independent prognostic genes were analyzed using the single-cell atlas of LIHC and verified in LIHC cells by carrying out in vitro assays. Wound healing and transwell assays were carried out to measure the abilities of LIHC cells to migrate and invade.

## 2. Material and Methods

### 2.1. Collection and Preprocessing of Data

Clinical data of LIHC in FPKM format were collected from TCGA database. Samples without survival time or status were deleted while ensuring that the remaining samples had a survival time longer than 0 day. Finally, the protein-coding genes were collected from 365 LIHC samples. TCGA–LIHC cohort served as a training set.

The ICGC-LIRI-JP dataset was collected by accessing HCCDB18 database (http://lifeome.net/database/hccdb/home.html). A sum of 203 LIHC samples were acquired by screening samples with survival time > 0 day. ICGC-LIRI-JP dataset was utilized as the validation set.

GSE149614 (https://www.ncbi.nlm.nih.gov/geo/query/acc.cgi?acc=GSE149614) with single-cell data was downloaded from the GEO database and primary tumor and nontumor samples were selected based on the clinical data. The library construction platform was 10 × Genomics, and the single-cell RNA sequencing (scRNA-seq) platform was Illumina NovaSeq6000 [24].

### 2.2. Weighted Gene Co-Expression Network Analysis (WGCNA)

Key SRRG-related modules in LIHC were identified employing the WGCNA R package [25]. First, SRRGs were collected from the gene set enrichment analysis (GSEA) “GOBP RESPONSE TO STARVATION” in the mSigDB. The SRRG score was calculated utilizing the GSVA R package [26]. Then, the pickSoftThreshold function was utilized to decide the soft threshold (*β*). Co-expression gene modules were identified by performing hierarchical clustering under the criterion of minModuleSize = 60. Next, the relationship between each module and SRRG score was analyzed using Spearman method to select critical modules with the highest correlation coefficient for further investigation.

### 2.3. Identification of Glycolysis-Related SRRGs

A sum of 326 glycolysis-related genes (GRGs) from the mSigDB gene set were collected. Then, single-sample GSEA (ssGSEA) was conducted to calculate the ssGSEA score for GRGs using the GSVA R package [27] and those GRGs with *p* < 0.05 and |*r*| > 0.2 were filtered by Pearson correlation analysis. The glycolysis-related SRRGs were obtained by taking the intersection between the GRGs and key module genes.

### 2.4. Functional Enrichment Analysis

Gene ontology (GO) and Kyoto Encyclopedia of Genes and Genomes (KEGG) [28–30] enrichment analysis was conducted in the clusterProfiler R package (*p* < 0.05) [31] based on the glycolysis-related SRRGs.

### 2.5. Development and Verification of a RiskScore

Prognostic glycolysis-related SRRGs in LIHC were filtered by univariate Cox regression analysis. Gene range was compressed by LASSO Cox regression analysis with the glmnet R package [32] and the model was further optimized by tenfold cross validation. Independent prognostic genes and corresponding gene coefficients were obtained and calculated by multivariate Cox regression analysis. The following formula [33] was established:

  $$\begin{array}{c} \text{RiskScore}=\sum \beta i\times ExPi. \end{array}$$

*β* and *i* refer to the gene coefficient in Cox regression model and the gene expression, respectively.

Patients in TCGA–LIHC cohort were assigned by the median RiskScore value into low- and high-risk groups, followed by performing Kaplan–Meier (K–M) survival analysis for the two risk groups. Receiver operating characteristic (ROC) curve was plotted by the timeROC R package [34] to test the performance of the RiskScore model. The model robustness was confirmed using ICGC-LIRI-JP dataset.

### 2.6. Immune Cell Infiltration Analysis

The infiltration of various immune cells in the TME of LIHC was evaluated applying four algorithms including ESTIMATE [35], TIMER (http://cistrome.org/TIMER), ssGSEA, and MCP-counter in R package [36]. Correlation analysis between the RiskScore, 10 types of immune cells and independent prognostic genes was performed employing Spearman method. The expressions of eight types of immune checkpoint genes in the two risk groups were compared.

### 2.7. GSEA

Biological pathways and molecular mechanisms related to the prognostic differences in different risk groups in TCGA–LIHC cohort were analyzed by performing GSEA using the gseKEGG function in the clusterProfiler R package [37], with a FDR < 0.05 indicating a significant enrichment. Then, the top 10 pathways showing the highest normalized enrichment score (NES) in the two risk groups were selected.

### 2.8. Establishment of a Nomogram and Verification

Distribution of the RiskScore among clinicopathological features, including grade, gender, age, and AJCC stage was compared in TCGA–LIHC cohort. Next, independent prognostic factors were determined by conducting univariate and multivariate Cox regression analysis. A nomogram incorporating the RiskScore and clinical characteristics was developed to further improve the survival and risk prediction for LIHC patients [38]. The accuracy and reliability of the nomogram were reflected by plotting calibration curve and decision curve analysis (DCA).

### 2.9. Analysis of Single Cell Profiles

Here, we aimed to clarify the expression characteristics of independent prognostic genes in different cell types and their distribution in the tumor microenvironment by single-cell analysis. The scRNA-seq analysis was performed on GSE149614 dataset to analyze the expressions of independent prognostic genes. The CreateSeuratObject function in Seurat *R* package [24] was used to set up the Seurat object to filter cells with gene counts between 200 and 7000 and mitochondrial gene ratio <10%. After using the NormalizeData function for data normalization, dimensionality reduction was achieved by principal components analysis (PCA) and batch effects were removed by Harmony R package [39]. Next, UMAP analysis was conducted using the RunUMAP function for further dimensionality reduction. Cells were clustered employing FindNeighbors and FindClusters functions (dims = 1:30 and resolution = 0.1). Finally, the cell types were annotated based on the marker genes provided by CellMarker2.0 database.

### 2.10. Cell Culture and Transfection

The transformed human liver epithelial-2 (THLE-2; IM-H473), LIHC cell line Huh7 (IM-H040) were acquired from the Immocell biotechnology Co., Ltd (Xiamen, China). The cells were cultured in DMEM (YaJi, DH101) with 10% fetal bovine serum (FBS, YaJi, 10099141C) and stored at 37°C in 5% CO_2_. Based on the descriptions of the manufacturer, Lipofectamine 2000 (Invitrogen, USA) was applied to transfect the small interfering (si) RNA of *SLC2A1* (si-*SLC2A1#1*, target sequence: 5′-GGCATCAACGCTGTCTTCTATTA-3′ and si-*SLC2A1#2*, target sequence: 5′-TGGCTTTGTGGCCTTCTTTGAAG-3′, Sangon, China) [40].

### 2.11. Quantitative Real-Time PCR (qRT-PCR)

Total RNA of THLE-2 and Huh7 cells was isolated applying the Trizol reagent (15596026, BSZH Scientific Inc., China) and then, reverse-transcribed to cDNA with Hieff NGS ds-cDNA Synthesis Kit (13488ES24, Yeasen, China). Subsequently, utilizing Hieff qPCR SYBR Green Master Mix (11201ES50, Yeasen, China), qRT-PCR amplification was used to measure the mRNA expressions of independent prognostic genes. The operating procedures were as follows: at 95°C for 5 min, 40 cycles at 95°C for 10 s, and at 60°C for 30 s. Supporting Information 2: Table S1 lists the primer sequences used for qRT-PCR. The 2^−*ΔΔ*CT^ method was employed to calculate the gene expressions, with *GAPDH* as an internal reference [41–43].

### 2.12. Cell Counting Kit-8 (CCK-8) Assay

LIHC cells at the logarithmic growth phase were plated into a 96-well plate at the density of 1 × 10^4^ cells/well and incubated at 37°C with 5% CO_2_ for 0, 24, 48, and 72 h. Subsequently, each well was supplemented with 10 μL of CCK-8 solution (Dojindo Molecular Technique, Inc.) to further incubate the samples for 2 h at 37°C. The absorbance at 450 nm was detected and the final results were averaged from three separate experiments.

### 2.13. Wound Healing Assay

The role of *SLC2A1* silencing in LIHC cell migration was examined by carrying out would healing experiment [44]. LIHC cells at a density of 3 × 10^5^ cells/well were seeded into the six-well plates. After scratching the cells with a sterilized pipette tip, the cells were then further incubated in serum-deleted medium for 48 h. Finally, an inverted microscope (CKX53, Olympus, Japan) was employed to capture the images at 0 and 48 h, and the ImageJ was utilized to calculate the wound closure (%) of LIHC cells.

### 2.14. Transwell Assay

The effect of *SLC2A1* silencing on LIHC cell invasion was analyzed transwell assay [45]. The Matrigel (0827045, sojubio, China) was used to precoat the upper transwell chamber (8.0 μm, 3422, Corning, USA), while DMEM with 10% FBS was added into the lower chamber. Next, the transfected LIHC cells (1 × 10^5^ cells/well) were added into the upper chamber with nonserum medium and cultivated at 37°C for 24 h. Afterwards, the cells invading into the lower chamber were fixed using 4% paraformaldehyde (ZY-131282, Zeye, Shanghai, China) and dyed by 0.1% crystal violet (mlsw-1800, mlBio, Shanghai, China). Finally, the cells were quantified under an inverted microscope [46].

### 2.15. Statistical Analysis

Bioinformatic analyses were completed in R language (version 3.6.0). Differences between two continuous variable groups were compared utilizing Wilcoxon rank test. Survival differences between different groups was compared by log-rank test. Statistical data were presented in mean ± standard deviation and analyzed by SPSS20.0. A *p* < 0.05 signified a statistical significance.

## 3. Results

### 3.1. Key Gene Modules Associated With SRRGs in LIHC Were Identified by WGCNA

**Figure 1:**
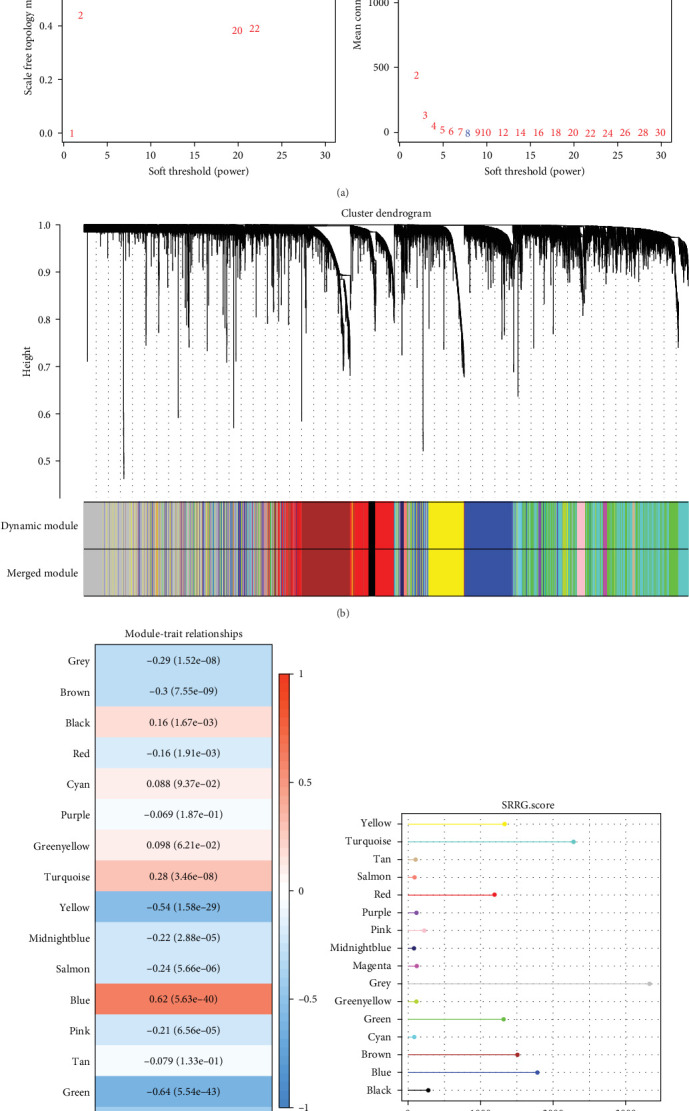
Identification of key gene module associated with starvation response-related genes (SRRG) in LIHC by WGCNA. (A) Scale-free fitting index analysis of soft threshold (power). (B) Cluster dendrogram of hierarchical clustering. (C) Module-trait relationships between each module and SRRG score. (D) Number of genes of each module.

Critical gene modules related to SRRG score in LIHC were identified by WGCNA under a soft threshold of as *β* = 8 (Figure 1A) to ensure a scale-free nature of the network. A total of 16 co-expression modules were classified by hierarchical clustering (Figure 1B). Module-trait relationship heatmap displayed that the blue module was most closely correlated with SRRG score (*r* = 0.62; Figure 1C), therefore, the genes in this module were used for subsequent analysis. The gene number in each module was visualized into lollipop plot (Figure 1D).

### 3.2. Functional Enrichment Analysis on the Glycolysis-Related SRRGs

**Figure 2:**
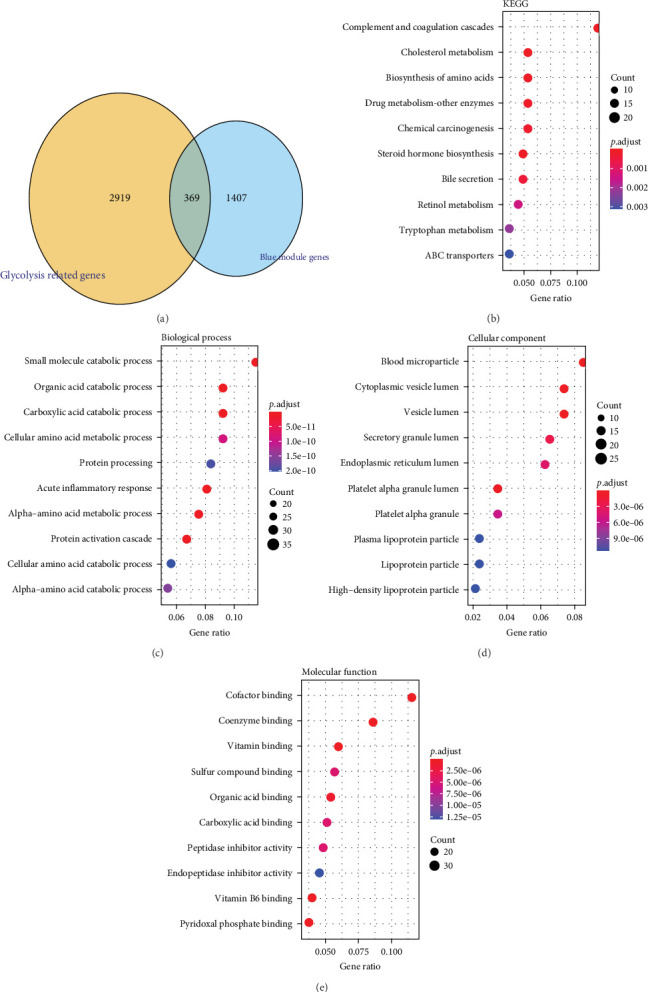
Functional enrichment analysis of the glycolysis-related SRRG. (A) Venn diagram of glycolysis-related genes (GRG) and blue module genes. (B) KEGG enrichment pathways of glycolysis-related SRRG. (C) GO enrichment terms in biological process (BP). (D) GO enrichment terms in cellular component (CC). (E) GO enrichment terms in molecular function (MF).

A total of 369 glycolysis-related SRRG were present in the intersection of the GRGs and blue module genes (Figure 2A). KEGG enrichment results showed that the glycolysis-correlated SRRGs were more enriched in the metabolism-correlated pathways, including cholesterol metabolism, retinol metabolism, drug metabolism-other enzymes, and tryptophan metabolism (Figure 2B), which may be involved in the progression of LIHC. GO enrichment results demonstrated that in the BP category, the glycolysis-related SRRGs were mainly enriched in the amino acid metabolic terms, including cellular amino acid metabolic process, alpha-amino acid metabolic process, and carboxylic acid catabolic process (Figure 2C). In the CC category, the GO terms of platelet alpha granule lumen, endoplasmic reticulum lumen, cytoplasmic vesicle lumen, secretory granule lumen, et cetera were significantly enriched (Figure 2D). In the MF category, the GO terms such as coenzyme binding, vitamin binding, organic acid binding, and carboxylic acid binding were notably enriched (Figure 2E). These pathways may influence the occurrence and development of LIHC.

### 3.3. A RiskScore Model Based on the Five Independent Prognostic Genes Was Established and Validated

**Figure 3:**
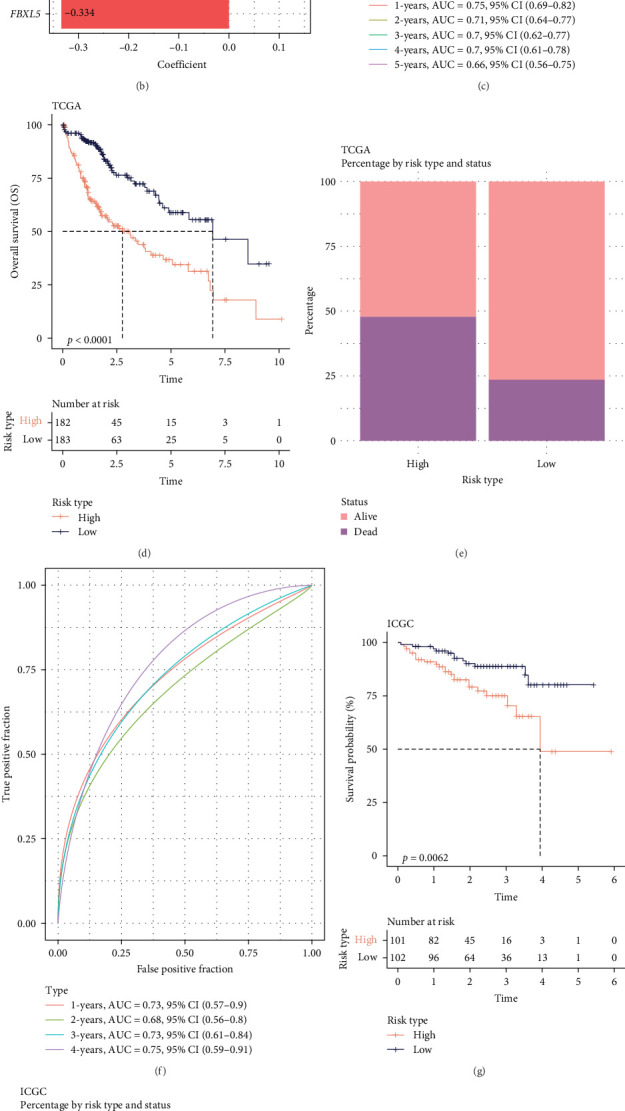
A RiskScore model developed based on independent prognostic genes and validation of the model. (A) LASSO Cox regression analysis to reduce gene range. (B) Randomforest plot of multivariate Cox regression analysis. (C) ROC curve of RiskScore model in TCGA-LIHC cohort. (D) Kaplan–Meier (K–M) curve of overall survival (OS) between different risk groups in TCGA-LIHC cohort. (E) Percentage of survival status between different risk groups in TCGA-LIHC cohort. (F) ROC curve of RiskScore model in ICGC-LIRI-JP dataset. (G) K–M curve of survival probability between different risk groups in ICGC-LIRI-JP dataset. (H) Percentage of survival status between different risk groups in ICGC-LIRI-JP dataset. (I, J) Expression levels of independent genes for LIHC prognosis in TCGA-LIHC and ICGC-LIRI-JP datasets.

Prognostically significant genes in LIHC were selected from the 369 glycolysis-related SRRGs by univariate and LASSO Cox regression analysis, and then, the model was refined using tenfold cross validation (Figure 3A). Two protective genes (*FBXL5* and *PON1*) and three risk genes (*TFF2*, *TBC1D30*, and *SLC2A1*) were determined as the most significant genes for LIHC prognosis (Figure 3B). Afterwards, based on the five independent prognostic genes, a RiskScore model was formulated as follow: RiskScore = (−0.334 × FBXL5) + (−0.09PON1) + (0.138 × SLC2A1) + (0.09 × TBC1D30) + (0.02 × TFF2).

The LIHC patients were divided by the median RiskScore value into high- and low-risk groups. The prediction of the RiskScore model in TCGA-LIHC cohort was reliable, with 1-year AUC of 0.75, 2-year AUC of 0.71, 3-year AUC of 0.7, 4-year AUC of 0.7, and 5-year AUC of 0.66 (Figure 3C). According to the K–M curve, overall survival (OS) and clinical outcomes in high-risk group in TCGA-LIHC cohort were unfavorable (Figure 3D). High-risk group had more death cases in TCGA–LIHC cohort (Figure 3E). Furthermore, the robustness of RiskScore model in ICGC-LIRI-JP dataset was verified, which was in accordance with the results in training set (Figure 3F–H). These results highlighted the potential value of the RiskScore model in the prognostic prediction for LIHC patients.

Moreover, the expressions of the five independent prognosis genes in TCGA-LIHC and ICGC-LIRI-JP datasets were visualized into heatmaps. It could be observed that *FBXL5* and *PON1* were high-expressed in low-risk group, whereas high-risk group had a high expression of *TFF2*, *TBC1D30*, and *SLC2A1* (Figure 3I,J).

### 3.4. Immune Cell Infiltration in Different Risk Groups

**Figure 4:**
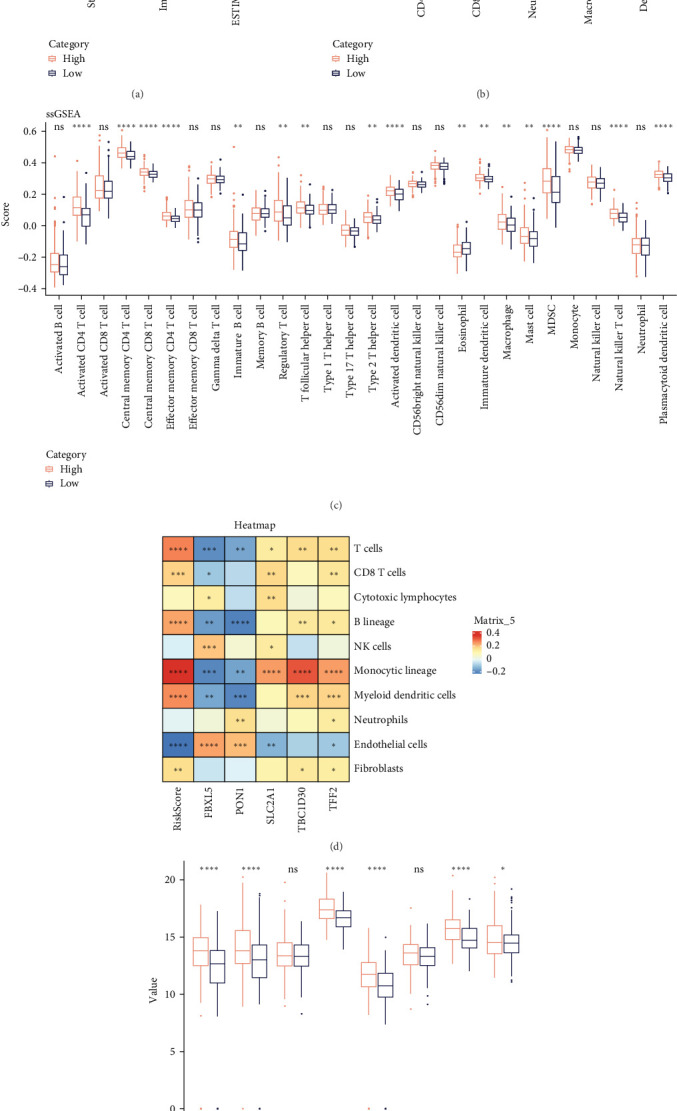
Immune cell infiltration between different risk groups. (A–C) Infiltration levels of immune cells between low- and high-risk groups calculated by ESTIMATE, TIMER, and ssGSEA algorithms. (D) Correlation between independent prognostic genes, RiskScore and infiltration levels of immune cells calculated by MCP-counter. (E) Expressions of immune checkpoint genes in the risk groups. *⁣*^*∗∗∗∗*^ means *p* < 0.0001; *⁣*^*∗∗∗*^ means *p* < 0.001; *⁣*^*∗∗*^ means *p* < 0.01; *⁣*^*∗*^ means *p* < 0.05; ns means not significant.

Analysis on the infiltration of immune cells in the TME of LIHC showed that ImmuneScore and ESTIMATEScore in high-risk patients were notably higher than those in low-risk group (Figure 4A). TIMER analysis demonstrated that high-risk group had higher infiltration of macrophages, neutrophils, dendritic cells, CD8 T cells, CD4 T cells, and B cells than the low-risk group (Figure 4B). The ssGSEA revealed that the infiltration of most immune cells including natural killer (NK) T cells, mast cells, T follicular helper cells, immature B cells, regulatory T cells (Tregs), and activated CD4 T cells were noticeably higher in high-risk group (Figure 4C). MCP-counter analysis showed that the infiltration of T cells, monocytic lineage, myeloid dendritic cells, CD8 T cells, and B lineage was positively correlated with the RiskScore (Figure 4D). These findings suggested that high-risk LIHC patients had higher immune cell infiltration and expressions of immune checkpoint genes (*CTLA4*, *PDCD1*, *LGALS9*, *CD80*, *HAVCR2*, and *LAG3*; Figure 4E), indicating that these patients exhibited stronger immune escape ability and worse prognosis.

### 3.5. GSEA on Different Risk Groups

**Figure 5:**
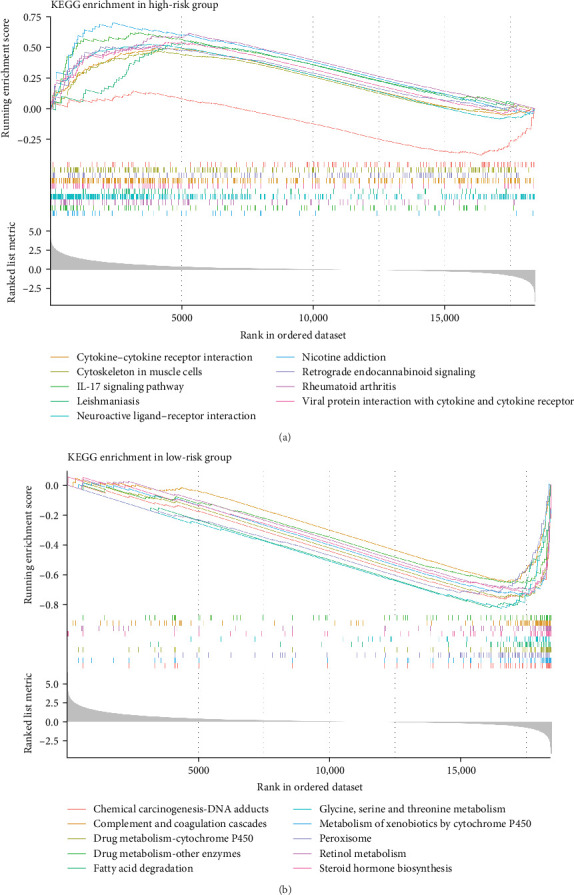
GSEA of different risk groups in TCGA-LIHC cohort. (A) KEGG enrichment pathways in high-risk group. (B) KEGG enrichment pathways in low-risk group.

In the two risk groups in TCGA–LIHC cohort, KEGG enrichment analysis revealed that cytokine–cytokine receptor interaction and IL–17 signaling pathway were significantly enriched in the high-risk group (Figure 5A), and that low-risk group had markedly enriched serine and threonine metabolism, glycine, fatty acid degradation, and metabolism of xenobiotics by cytochrome P450 (Figure 5B). This indicated that the TME of LIHC patients in low-risk group may have unique biological functions in energy metabolism and detoxification.

### 3.6. Development of a Nomogram With RiskScore and AJCC Stage

**Figure 6:**
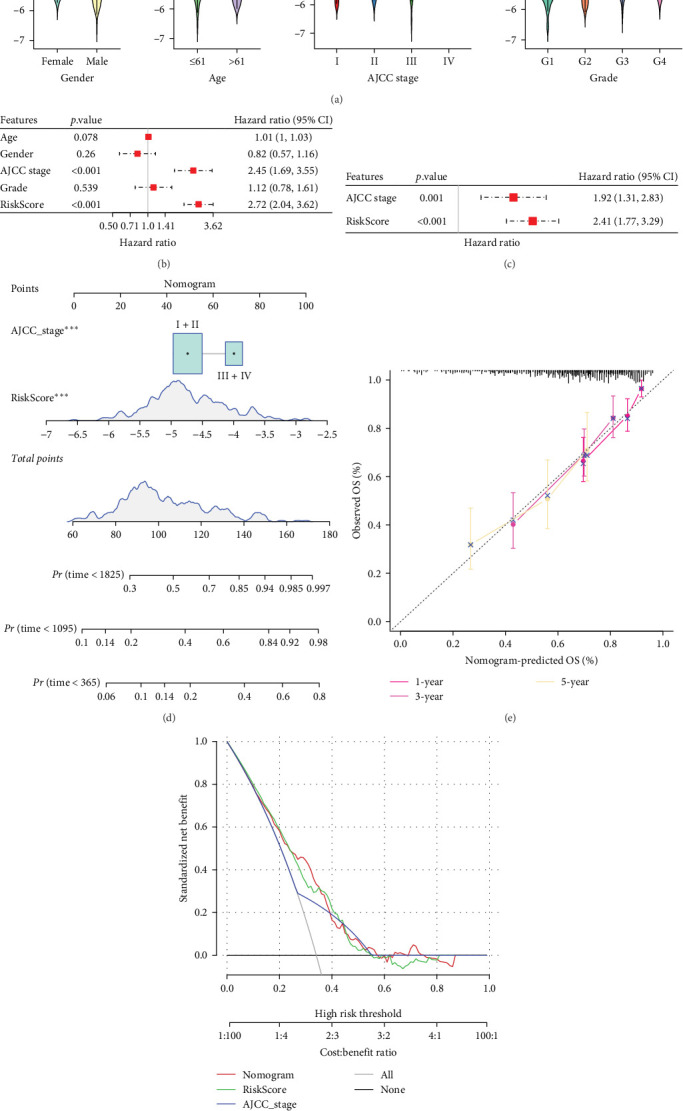
Construction of nomogram and validation. (A) Distribution of RiskScore in different clinical characteristics such as gender, AJCC stage, grade, and age. (B, C) Univariate and multivariate Cox regression analysis of RiskScore and clinical features. (D) Nomogram model constructed by combining RiskScore and AJCC stage; *⁣*^*∗∗∗*^ means *p* < 0.001. (E) Calibration curved of the nomogram for 1, 3, and 5 year(s). (F) Decision curve analysis (DCA) of nomogram.

To further explore the relationship between RiskScore and patients' clinicopathologic characteristics, first, we found, based on the TCGA-LIHC cohort, that RiskScore would be higher in women. In addition, RiskScore would be higher in patients with higher AJCC stage, higher Grade, and age ≤61 years (Figure 6A). The RiskScore and AJCC stage were verified as independent factors for LIHC prognosis, according to the results of the univariate and multivariate Cox regression analysis (Figure 6B,C). Then, a nomogram was established by combining the two factors (Figure 6D). The predicted calibration curves of the nomogram at 1, 3, and 5 year(s) were close to the standard curves (Figure 6E), indicating a strong predictive performance of the nomogram. According to DCA, the net benefits of the nomogram and RiskScore were notably higher the extreme curve (Figure 6F), which further supported that the nomogram was reliable.

### 3.7. The Expressions of Independent Prognostic Genes in Single-Cell Atlas of LIHC

**Figure 7:**
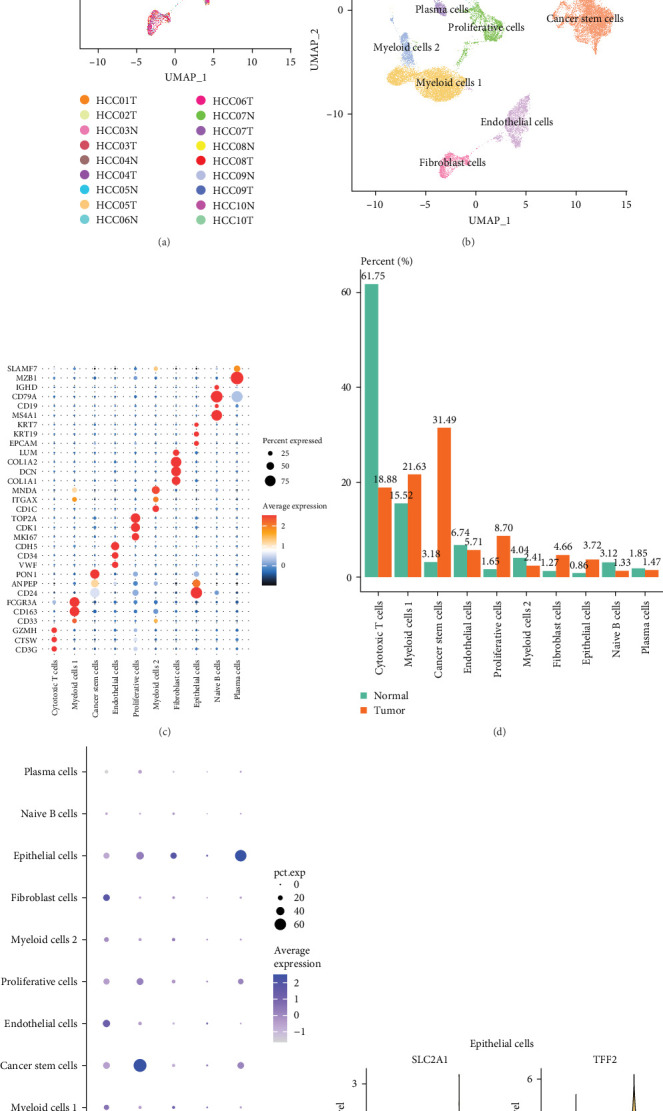
Single-cell atlas of LIHC. (A) UMAP plot of 18 primary tumor and adjacent nontumor liver samples. (B) UMAP dimensionality reduction plot of single-cell clustering and annotation. (C) Bubble plot of marker genes in each cell cluster. (D) Percentage of each cell cluster in tumor and nontumor groups. (E) Expression levels of independent prognostic genes in each cell cluster of tumor group. (F) Expressions of *SLC2A1* and *TFF2* in epithelial cells.

A total of 18 liver samples of primary tumor and adjacent nontumor were subjected to single-cell clustering analysis. All the cells were annotated into 10 cell clusters (Figure 7A, B), including Cytotoxic T cells, epithelial cells, naive B cells, proliferative cells, myeloid cells 2, cancer stem cells, plasma cells, and myeloid cells 1. From the bubble plot of the marker genes in the cell clusters, it could be observed that some marker genes (*PON1*, *ANPEP*, and *CD24*) expressed in cancer stem cells were also expressed in epithelial cells (Figure 7C), suggesting that epithelial cells had the features of tumor cells in LIHC progression or the potential to transform into cancer cells. Moreover, the proportion of cytotoxic T cells in tumor group (18.88%) was markedly lower than that in non-tumor group (61.75%) but the proportion of cancer stem cells in tumor group (31.49%) was notably higher than that in non-tumor group (3.18%) (Figure 7D). In addition, the five independent prognostic genes were mainly expressed in cancer stem cells and epithelial cells (Figure 7E), in particular, the expressions of *SLC2A1* and *TFF2* in epithelial cells of tumor group were notably higher than those of nontumor group (Figure 7F).

### 3.8. Validation Analysis Based on the Key Markers Screened by SRRGs

**Figure 8:**
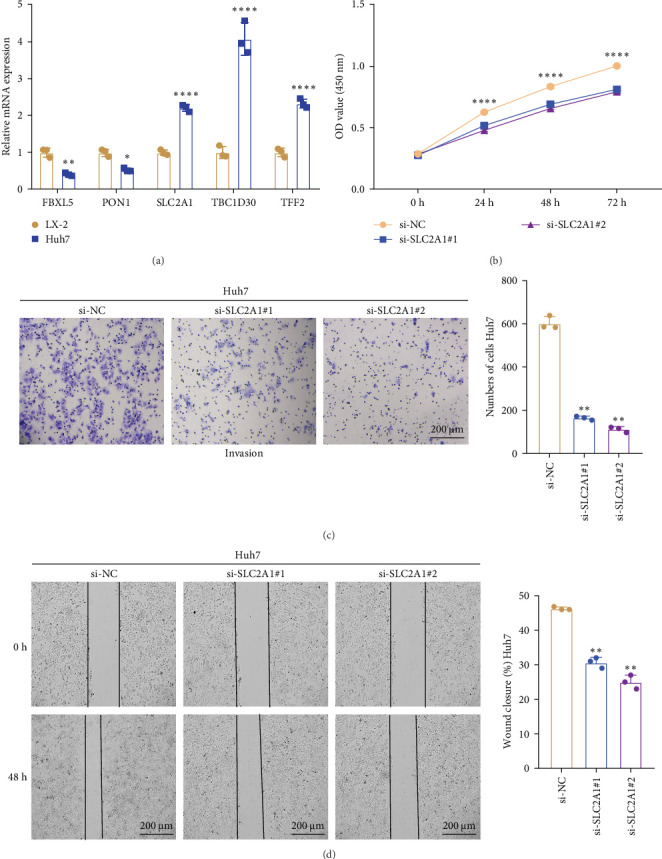
The in vitro validation by LIHC cells. (A) qRT-PCR detecting the relative mRNA expressions of five prognostic SRRGs. (B) CCK-8–based assay to assess the effect of silencing *SLC2A1* on LIHC cell viability. (C) Transwell assay assessing the impact of *SLC2A1* silencing on cell invasion of Huh7 cells. (D) Wound healing assay evaluating the effect of *SLC2A1* silencing on cell migration of Huh7 cells. All procedures were repeated three times independently. *⁣*^*∗∗∗∗*^ means *p* < 0.0001; *⁣*^*∗∗∗*^ means *p* < 0.001; *⁣*^*∗∗*^ means *p* < 0.01; and *⁣*^*∗*^ means *p* < 0.05.

The expressions of the five genes in different stages and grades in the TCGA database were compared. The expression of *PON1* in the late stage (Stage III + IV) was significantly lower than in the early stage, whereas the expressions of *TFF2* and *SLC2A1* were remarkably higher in the late stage than in the early stage (Stage I + II; Supporting Information 3: Figure 2A). Additionally, *PON1* and *FBXL5* had significantly higher expression in low-grade tumors (G1 + G2) than in high-grade tumors (G3 + G4), whereas high-grade tumors (G3 + G4) had noticeably higher level of *SLC2A1* than in low-grade tumors (G1 + G2; Supporting Information 3: Figure 2B). Next, according to qRT-PCR test, *FBXL5* and *PON1* were low-expressed but *TFF2*, *TBC1D30*, and *SLC2A1* were high-expressed in the LIHC cells Huh7 (Figure 8A). As *SLC2A1* was a risk factor expressed at more a higher level in LIHC cells, we further explored the potential biological functions of *SLC2A1* on LIHC cells. The cell viability of Huh7 was significantly suppressed in comparison to the control cells after silencing *SLC2A1* (Figure 8B). Further, the transwell assay revealed that *SLC2A1* silencing notably lowered the number of invaded LIHC cells (Figure 8C). Wound healing assay also showed that the silencing of *SLC2A1* markedly lowered the wound closure rate of LIHC cells (Figure 8D). These data suggested that *SLC2A1* played a critical role in the progression of LIHC.

## 4. Discussion

LIHC shows high metastasis and recurrence rates worldwide [47, 48]. Rapidly proliferating tumors usually experience nutrient or energy starvation, but neoplastic cells can cope with the cytotoxicity of metabolic stress through molecular adaptations [49]. Starvation response functions crucially in the initiation of LIHC and its development [50]. Lei et al. [51] developed a starvation-related 9-mRNA signature for predicting the progression and prognostic results of LIHC. In our present study, we identified five independent prognostic SRRGs (*FBXL5*, *PON1*, *TFF2*, *TBC1D30*, and *SLC2A1*) to create a RiskScore model, which accurately predicted the prognostic outcomes for patients with LIHC. High-risk patients had a low survival chance and a poor clinical outcome, interestingly the two risk groups of patients exhibited different immune cell infiltration and biological functions. Meanwhile, by integrating RiskScore with AJCC stage, a nomogram with a strong predictive performance was established. These findings indicated that the SRRG signature could be utilized as a reliable prognostic signature for LIHC.

The five independent prognostic SRRG in LIHC consisted of two protective genes (*FBXL5* and *PON1*) and three risk genes (*TFF2*, *TBC1D30*, and *SLC2A1*). *FBXL5*, a subunit of E3 ubiquitin ligase, exerts a critical effect on regulating the metastasis and chemoresistance of tumorous cells via targeting various substrates for ubiquitin-mediated disruption [52]. *FBXL5* deficiency in the liver promotes the carcinogenesis of LIHC by disturbing cellular iron homeostasis in a mouse model [53]. A low mRNA expression of *FBXL5* is predictive of worse recurrence-free survival, disease-specific survival and poor prognosis of LIHC patients [54]. *PON1*, a calcium-dependent esterase, is principally synthesized by hepatocytes in liver and then released into the plasma [55]. *PON1* has antioxidant defense effects and serum *PON1* is involved in cell injury and chronic inflammation of liver [56]. Multiple researches showed that *PON1* exhibits a strong diagnostic potential in distinguishing alpha-fetoprotein (AFP)-negative LIHC patients from those with AFP cirrhosis [57]. Downregulated *PON1* is an indicator of a low survival rate for LIHC patients [58]. *TFF2*, a member of trefoil factor family, is expressed mainly in the gastrointestinal epithelium to protect mucosa and promote epithelial repair [59]. It was reported that *TFF2* fulfills an antitumor function by restraining the growth and invasion and enhancing the apoptosis of cancer cells in cholangiocellular carcinoma [60], gastric cancer [61], and pancreatic cancer [62]. *TBC1D30* belongs to TBC1-domain family member 30 [63]. *TBC1D30* has been identified as one of the putative biomarkers for temozolomide resistance in patients with primary glioblastoma multiforme [64]. *SLC2A1* is an important solute carrier that facilitates glucose to penetrate target cells in the Warburg effect [65]. Upregulated *SLC2A1* in LIHC tumor tissue is correlated with the clinical stage and prognosis of patients [66]. Moreover, a complex relationship between a high *SLC2A1* expression and drug sensitivity of LIHC patients has been reported [67]. In our current study, *FBXL5* and *PON1* were low-expressed but *TFF2*, *TBC1D30*, and *SLC2A1* were high-expressed in high-risk group. According to the result of scRNA-seq analysis, the five prognostic SRRGs were mainly expressed in cancer stem cells and epithelial cells, in particular, the expressions of *SLC2A1* and *TFF2* in epithelial cells of tumor group were notably higher than nontumor group. In addition, the migration and invasion of LIHC cells were suppressed by *SLC2A1* silencing. Thus, the five SRRGs could be considered as therapeutic targets and prognostic biomarkers for LIHC.

Furthermore, immune cells in TME play indispensable roles in the occurrence, metastasis, and drug resistance of LIHC, greatly influencing the prognostic outcomes and immunotherapy efficacy of patients [68]. The present research observed that high-risk LIHC patients had higher infiltration of immune cells including T follicular helper cells, mast cell Tregs, CD8 T cells, NK T cells, immature B cells, CD4 T cells, and macrophages than in low-risk group and this was linked to poor outcomes. Tregs are one of the critical factors in maintaining immune tolerance and facilitating immune escape of tumors [69]. Macrophages are a type of key cells involved in steatosis-triggered LIHC progression [70]. In tumor development, mast cells not only induce tumor angiogenesis, migration, and invasion, but is also involved in adaptive immune responses [71]. Researchers also discovered that high immune cell infiltration of macrophages, CD4 T cells, CD8 T cells, and B cells shapes the immune inflammatory phenotype in LIHC [72]. Meanwhile, we observed the high-risk LIHC group with higher expressions of the immune checkpoint genes (*CTLA4*, *PDCD1*, *LGALS9*, *CD80*, *HAVCR2*, and *LAG3*) had a stronger immune escape ability [73]. In addition, GSEA showed that high-risk group had notably enriched IL-17 signaling pathway and cytokine–cytokine receptor interaction. Cytokines produced by tumor cells possess a cooperative effect with immune cells in TME and together greatly enhance tumor activity [74]. IL-17 signaling pathway is correlated with immunity and inflammation [75]. These metabolic pathways were all involved in the mechanism of LIHC progression. To conclude, the prognostic RiskScore model developed based on the five SRRGs could guide the immunotherapy and personalized treatment for LIHC patients.

However, some limitations in the present work should be pointed out. First, the LIHC samples used in the present work were all collected from the public databases and clinical tests are required to validate our findings. Second, the roles of SRRGs in LIHC progression have not been explored. Future studies are encouraged to perform in vivo and in vitro experiments to verify the clinical significance of the SRRG signature in LIHC.

## 5. Conclusion

To conclude, based on five SRRGs (*FBXL5*, *PON1*, *TFF2*, *TBC1D30*, and *SLC2A1*), the present research created a RiskScore model that can accurately predict the prognostic outcomes for LIHC. The high-risk LIHC group had a poorer clinical outcome, high immune cell infiltration rate, and high immune escape capacity, indirectly suggesting the development of an immunosuppressive for environment. The current five-SRRG signature has manifested its significant potential prognostic values in LIHC treatment.

## Data Availability

The datasets generated and/or analyzed during the current study are available in the GSE149614 repository (https://www.ncbi.nlm.nih.gov/geo/query/acc.cgi?acc=GSE149614).

## Nomenclature

AFP: Alpha-fetoprotein
AJCC: American Joint Committee on Cancer
AUC: Area under ROC curve
BP: Biological process
CC: Cellular component
DCA: Decision curve analysis
DMEM: Dulbecco's modified eagle's medium
ESTIMATE: Estimation of stromal and immune cells in malignant tumor tissues using expression data
FBS: Fetal bovine serum
FDR: False discovery rate
FPKM: Fragments per kilobase of transcript per million fragments mapped
GEO: Gene Expression Omnibus
GO: Gene Ontology
GRG: Glycolysis-related genes
GSEA: Gene set enrichment analysis
HCCDB18: Integrative molecular database of hepatocellular carcinoma
ICIs: Immune checkpoint inhibitors
KEGG: Kyoto Encyclopedia of Genes and Genomes
K–M: Kaplan–Meier
LASSO: Least absolute shrinkage and selection operator
LIHC: Liver hepatocellular carcinoma
MCP-counter: Microenvironment cell populations-counter
MF: Molecular function
mSigDB: Molecular signatures database
NES: Normalized enrichment scores
NK: Natural killer
OS: Overall survival
PCA: Principal components analysis
qRT-PCR: Quantitative real-time polymerase chain reaction
ROC: Receiver operating characteristic
scRNA-seq: Single-cell RNA sequencing
si: Small interfering
SRRG: Starvation response-related genes
ssGSEA: Single-sample gene set enrichment analysis
TCGA: The cancer genome atlas
TIICs: Tumor infiltrating immune cells
TIMER: Tumor immune estimation resource
TKIs: Tyrosine kinase inhibitors
TME: Tumor microenvironment
Tregs: Regulatory T cell
UMAP: Uniform manifold approximation and projection
WGCNA: Weighted gene co-expression network analysis.

## Supporting Information

Supporting Information 1 Figure S1. Analysis flow chart.

Supporting Information 2 Table S1. Primer sequences used in qRT-PCR.

Supporting Information 3 Figure S2. Based on the TCGA database to assess differences in expression of five LIHC markers (FBXL5, PON1, SLC2A1, TBC1D30, and TFF2) at different stages and grades.
