# Supplementary figures and images for: Development of a Starvation Response–Based Model and Its Application in Prognostic Assessment of Liver Hepatocellular Carcinoma
Source: Mediators Inflamm. 2025 Jul 7;2025:8828435. doi: 10.1155/mi/8828435 (PMC12259329; doi:10.1155/mi/8828435)

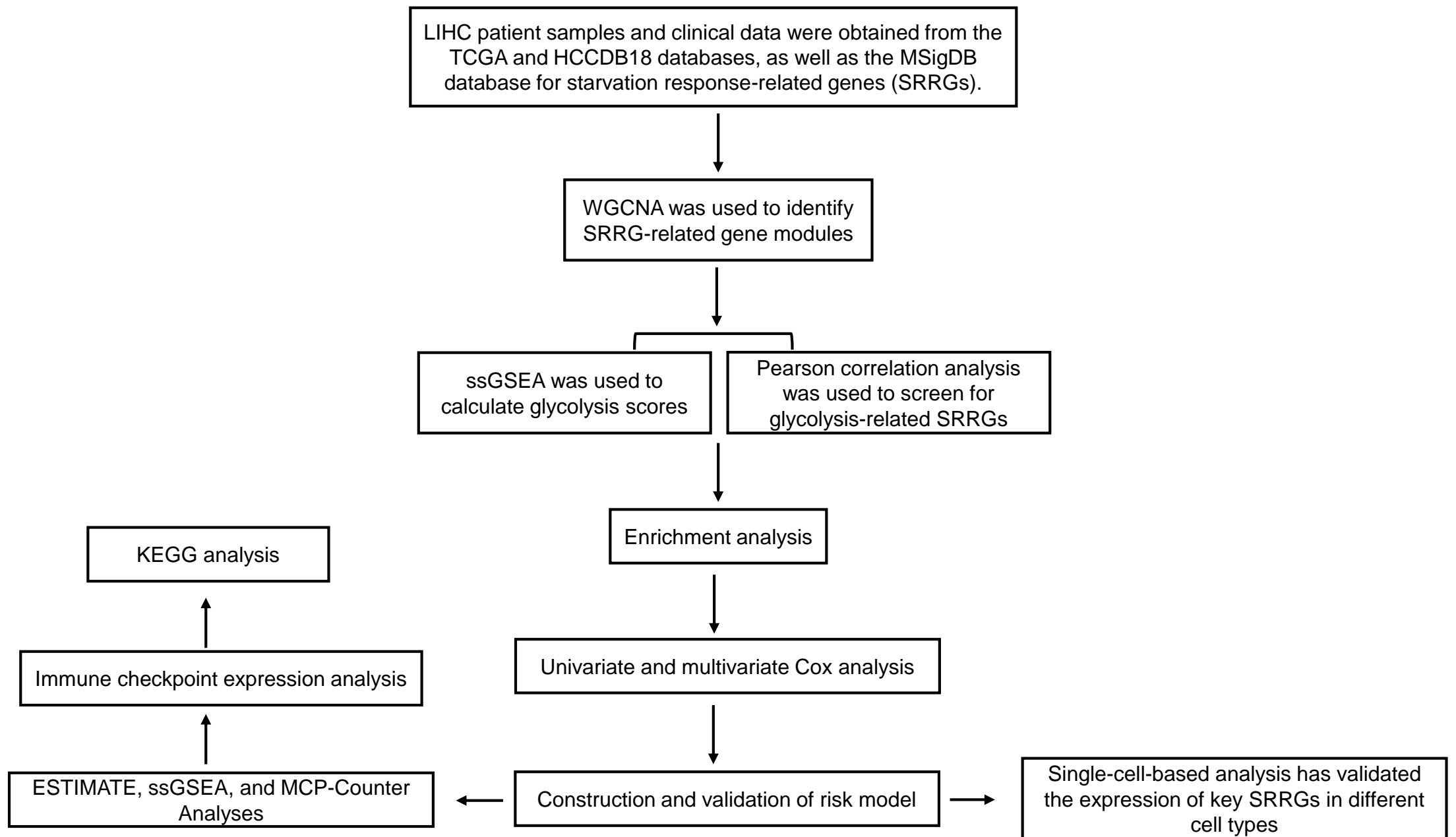

Supplement: Supporting Information 1 — Figure S1. Analysis flow chart. [file 8828435.f1.pdf]

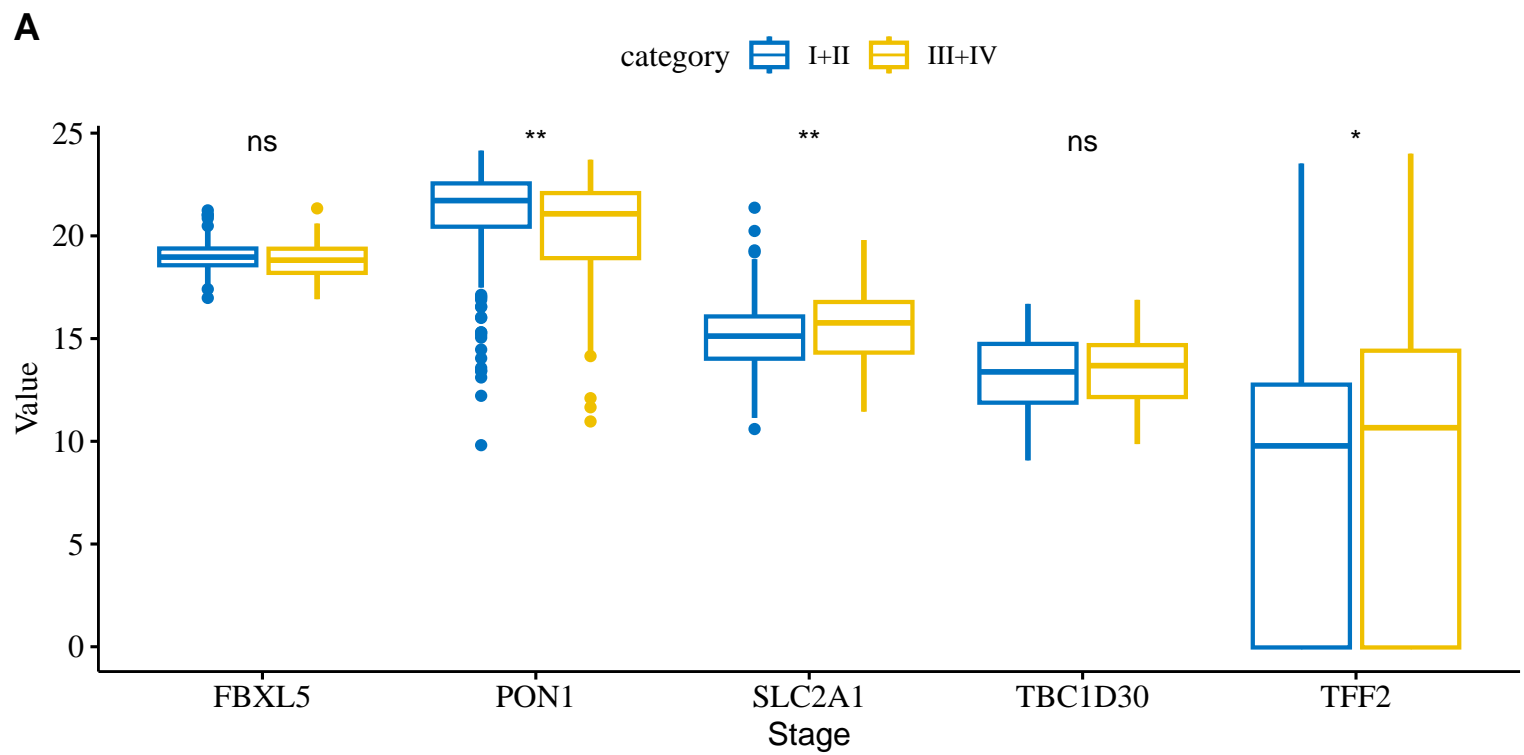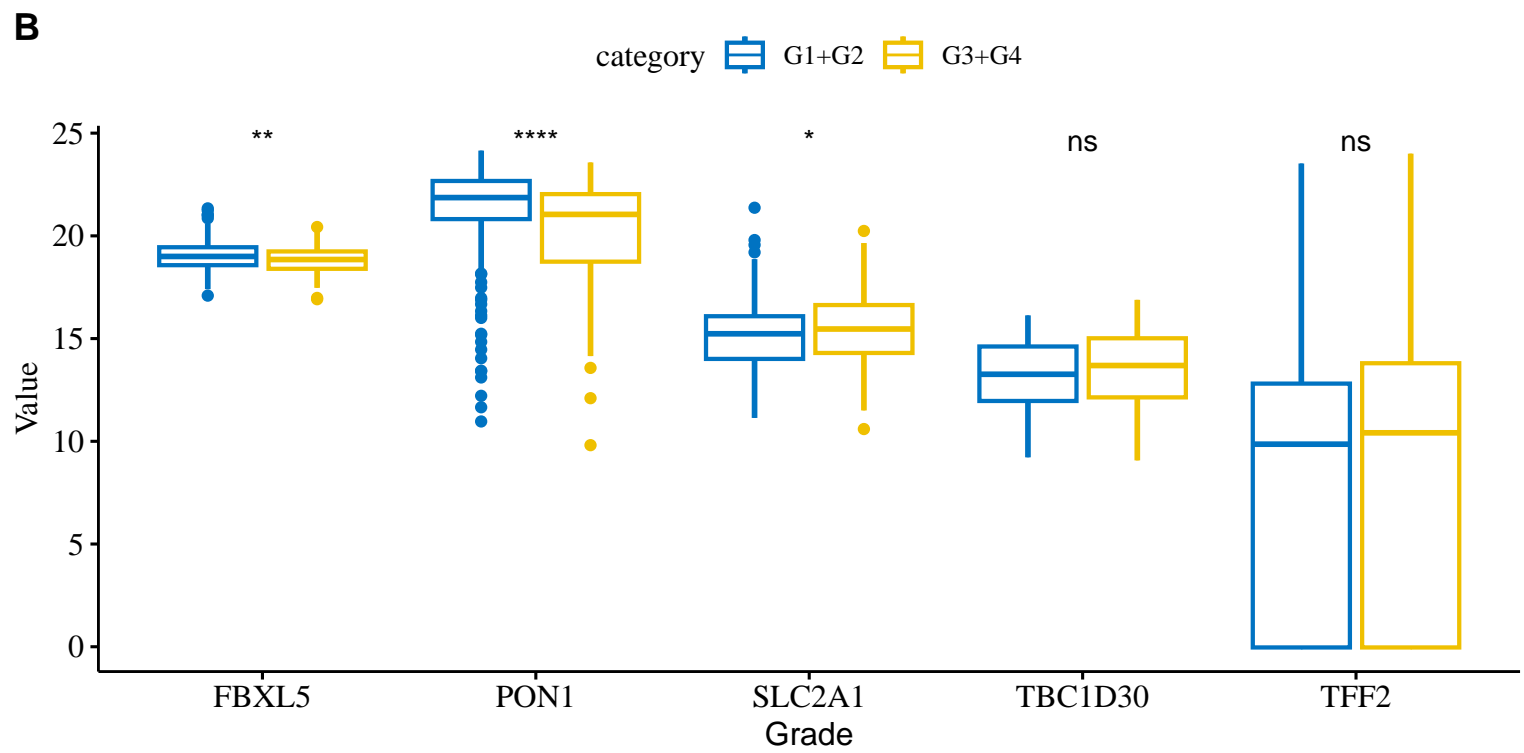

Supplement: Supporting Information 3 — Figure S2. Based on the TCGA database to assess differences in expression of five LIHC markers (FBXL5, PON1, SLC2A1, TBC1D30, and TFF2) at different stages and grades. [file 8828435.f3.pdf]
