# Supplementary material for: Development of a Starvation Response–Based Model and Its Application in Prognostic Assessment of Liver Hepatocellular Carcinoma
Source: Mediators Inflamm. 2025 Jul 7;2025:8828435. doi: 10.1155/mi/8828435 (PMC12259329; doi:10.1155/mi/8828435)
Supplement: Supporting Information 2 — Table S1. Primer sequences used in qRT-PCR. [file 8828435.f2.docx]

**Supplementary Table 1. Primer sequences used in qRT‑PCR**

| Gene | Primers (5’-3’) |
| --- | --- |
| *FBXL5* | Forward: CTTTCCTGAAGAAGTGGAC  Reverse: GGTTATACCTGTGGAGTGTT |
| *PON1* | Forward: TCTTGACCCCTACTTACAAT  Reverse: AAACCTGTGTCACTTTAGGT |
| *SLC2A1* | Forward: TGTCTTCTATTACTCCACGA  Reverse: TGAAGATGATGAAGACGTAG |
| *TBC1D30* | Forward: AGCAATGTGCTCAAGAAG  Reverse: CTTAGGATGATTTCTGAACC |
| *TFF2* | Forward: AGTGACCAGTGTTTTGACA  Reverse: CTTCAAAGATGAAGTTGGAG |
| *GAPDH* | Forward: CTACATGGTTTACATGTTCC  Reverse: CATACTTCTCATGGTTCACA |
